# Implementation of a lifestyle intervention for people with a severe mental illness (SMILE): a process evaluation

**DOI:** 10.1186/s12913-021-07391-3

**Published:** 2022-01-05

**Authors:** Florine S. Walburg, Johanna W. de Joode, Hella E. Brandt, Maurits W. van Tulder, Marcel C. Adriaanse, Berno van Meijel

**Affiliations:** 1 Department Health Sciences, Faculty of Science, Vrije Universiteit Amsterdam, Amsterdam Public Health research institute, Amsterdam, The Netherlands; 2 Department Human Movement Sciences, Faculty Behavioural and Movement Sciences, Vrije Universiteit Amsterdam, Amsterdam, The Netherlands; 3 Department of Health, Sports & Welfare, Research Group Mental Health Nursing, Inholland University of Applied Sciences, Amsterdam, The Netherlands; 4 Amsterdam UMC (VUmc), Department of Psychiatry, Amsterdam Public Health research institute, Amsterdam, The Netherlands; 5 Parnassia Psychiatric Institute, Parnassia Academy, The Hague, The Netherlands

**Keywords:** Process evaluation, Lifestyle intervention, Implementation, Nursing, Severe mental illness

## Abstract

**Background:**

Several interventions have been developed to improve physical health and lifestyle behaviour of people with a severe mental illness (SMI). Recently, we conducted a pragmatic cluster-randomised controlled trial which evaluated the effects of the one-year Severe Mental Illness Lifestyle Evaluation (SMILE) lifestyle intervention compared with usual care in clients with SMI. The SMILE intervention is a 12-month group-based lifestyle intervention with a focus on increased physical activity and healthy food intake. The aim of the current study was to explore the experiences of people with SMI and healthcare professionals (HCPs) regarding implementation feasibility of the SMILE intervention and the fidelity to the SMILE intervention.

**Methods:**

A process evaluation was conducted alongside the pragmatic randomized controlled trial. The experiences of clients and HCPs in the lifestyle intervention group were studied. First, descriptive data on the implementation of the intervention were collected. Next, semi-structured interviews with clients (*n* = 15) and HCPs (*n* = 13) were performed. Interviews were audiotaped and transcribed verbatim. A thematic analysis of the interview data was performed using MAXQDA software. In addition, observations of group sessions were performed to determine the fidelity to the SMILE intervention using a standardised form.

**Results:**

Ten out of 26 HCPs who conducted the group sessions discontinued their involvement with the intervention, primarily due to changing jobs. 98% of all planned group sessions were performed. Four main themes emerged from the interviews: 1) Positive appraisal of the SMILE intervention, 2) Suggestions for improvement of the SMILE intervention 3) Facilitators of implementation and 4) Barriers of implementation. Both clients and HCPs had positive experiences regarding the SMILE intervention. Clients found the intervention useful and informative. The intervention was found suitable and interesting for all people with SMI, though HCPs sometimes had to tailor the intervention to individual characteristics of patients (e.g., with respect to cognitive functioning). The handbook of the SMILE intervention was perceived as user-friendly and helpful by HCPs. Combining SMILE with daily tasks, no support from other team members, and lack of staff and time were experienced as barriers for the delivery of the intervention.

**Conclusion:**

The SMILE intervention was feasible and well-perceived by clients and HCPs. However, we also identified some aspects that may have hindered effective implementation and needs to be considered when implementing the SMILE intervention in daily practice.

**Supplementary Information:**

The online version contains supplementary material available at 10.1186/s12913-021-07391-3.

## Introduction

People with a severe mental illness (SMI) have an increased risk of developing cardiovascular disease [1–3]. This increased risk is primarily attributed to the high prevalence of cardiovascular risk factors, such as obesity, hypertension and dyslipidaemia [4], resulting in a life expectancy that is up to 20 years lower than the general population [5–8]. These cardiovascular risk factors are primarily the consequence of modifiable lifestyle factors, such as a sedentary lifestyle, unhealthy eating habits and smoking [9–12].

Several studies have addressed these modifiable cardiovascular risk factors in people with SMI in order to improve somatic health and life expectancy [13, 14]. However, there is still a gap between the available evidence regarding lifestyle interventions and the actual use of lifestyle interventions in daily practice [14–16]. To date, regular mental healthcare still lacks attention regarding lifestyle promotion, even though its importance has been recognized for decades [17]. To stimulate integration of lifestyle promotion in regular care for people with SMI, more knowledge and insight is needed on the delivery and implementation of lifestyle interventions [14].

From February 2018 to March 2020, the Severe Mental Illness Lifestyle Evaluation (SMILE) intervention was implemented among Flexible Assertive Community Treatment (FACT) teams in the Netherlands. The SMILE intervention focuses on healthy eating and exercising for clients with SMI. FACT-teams deliver long-term and flexible outpatient care for people with SMI, with the possibility to scale up or down the intensity of care on the basis of the client’s mental health status and care needs. The SMILE intervention consists of a one-year group-based lifestyle program and is based on the STRIDE intervention that was developed in the USA [18]. The STRIDE study found significant weight loss, where participants in the experimental condition lost 4.4 kg after 6 months and 2.6 kg after 12 months more than the patients in the control condition. For our study, some parts of the STRIDE intervention were slightly modified, for example more focus on portion control than on calorie counting and minor changes to better fit Dutch food habits, such as using Dutch food labels and referring to specific Dutch food habits [18, 19]. We believed that with these adaptations, the recognizability of the content of the intervention would improve for the patient population within FACT-teams. In addition to our focus on the effectiveness of the intervention program with respect to physical health of people with SMI, the results of which will be reported elsewhere, we assessed the feasibility to perform such an intensive lifestyle intervention for people with SMI in a real-world setting.

For this latter purpose, we collected descriptive data of the implementation process, followed by studying perceived barriers and facilitators regarding feasibility and implementation from the perspectives of both clients and HCPs. This information is vital because these barriers and facilitators have a great influence on the effectiveness of lifestyle interventions and form the basis for appropriate strategies for future implementation. The aim of this study was to explore the experiences of clients with SMI and HCPs regarding the feasibility and implementation of the SMILE intervention as well as the fidelity to the SMILE intervention.

## Methods

### Study design

A qualitative research design with semi-structured interviews in combination with observations of group sessions was used as part of a pragmatic cluster-randomized controlled trial (RCT) on implementation of a lifestyle intervention (SMILE) in Dutch mental health care compared with usual care. We used data-triangulation with the following data sources: interviews with both clients and HCPs, descriptive data from the RCT, and on-site observations performed by the researchers. The study was performed in accordance with the Declaration of Helsinki.

### SMILE intervention

The intervention was given by trained mental HCPs: mental health nurses, experts-by-experience, social workers, activity workers and psychologists. At least two HCPs from each FACT-team in the intervention group delivered the intervention. Prior to the start of the intervention, HCPs received a two-day training with the focus on healthy nutrition, physical activity, use of motivational interviewing techniques and becoming familiar with the SMILE handbook. The SMILE handbook was used to promote standardization of the intervention delivery. Clients could participate in the SMILE intervention if they had a minimum BMI of 27 and were 18 years or older. In the first 6 months (initial phase) group sessions took place weekly, and during the last 6 months (maintenance phase) monthly. Each group session started with a check-in, where successes and problems experienced during the past week were discussed. This was followed by discussing one or two specific topics scheduled for that session (appendix I). Afterwards, participants formulated their own personal goals for the upcoming week or month. There was also a 20-to-30-min workout in between or at the end of the session, which included walking with the group or indoor exercises. The SMILE intervention was delivered within 11 FACT- teams. The teams were located throughout different regions in The Netherlands. The methods and design of the SMILE study, including the content of the intervention, have been described previously [19].

### Participant and team selection of qualitative analysis

#### Interviews

The interviews were conducted with both clients and HCPs in order to obtain data on the feasibility of the SMILE intervention and on the implementation process. We used purposive sampling to include clients with a broad range of perspectives on the pre-specified topics [20]. Clients were selected on the basis of (1) attendance to group sessions, (2) weight change after 6 months, (3) gender and (4) diagnosis. We mainly selected clients for the interviews who fully completed the SMILE intervention; only one client was interviewed who dropped-out during the intervention period. Also, clients who experienced major challenges with the SMILE intervention were invited for the interview to obtain in-depth insight into barriers for implementation from the clients’ perspective. Clients were recruited through the HCPs involved in the intervention and asked if the researchers could contact them for an interview. HCPs were selected on the basis of their involvement in the implementation of the SMILE intervention, with a balanced representation of the disciplines involved: mental health nurse, expert-by-experience, social worker, activity worker and psychologist.

We included at least one client and one HCP from each of the 11 intervention teams. Clients and HCPs were informed about the process evaluation and asked if they were interested in participating in an interview. All but three clients who were approached for the interview agreed with participation. All HCPs approached agreed to be interviewed. Both clients and HCPs signed an informed consent form before the interview took place. All clients and HCPs received a 20-euro gift certificate card for participating in the interviews.

#### Observations

In addition to the interviews, in all participating intervention teams, on-site observations of SMILE group sessions were performed to assess fidelity to the intervention and interaction between clients and HCPs. For the purpose of these observations, one of the researchers joined a group session as an observer. We reached out to all intervention teams to ask if we could join and observe at least one session. The clients were always informed that one of the researchers would be joining as an observer.

### Data collection

#### Descriptive data

Total inclusion of clients in the intervention, attendance of clients in group sessions, dropout among HCPs (including reasons) and number of group sessions provided by HCPs were registered during the trial. These data were used as background information for the interviews conducted.

#### Interviews

Two topic lists for the interview guides (one for the interviews with clients and one for HCPs) were developed based on the RE-AIM framework [21, 22] and the Measurement Instrument for Determinants of Innovation (MIDI) [23] (see Appendix II for topic lists). RE-AIM assesses five dimensions of the process of implementation of an intervention: Reach, Efficacy, Adoption, Implementation, and Maintenance. Reach explores characteristics of study participants compared to the target population; Effectiveness refers to the impact of the SMILE intervention on the outcomes; Adoption assesses the proportion of the intervention that is adopted by HCPs; Implementation assesses the fidelity to the intervention and resources (e.g. time); Maintenance evaluates the sustainability of the implementation of the intervention on both the individual and organizational/setting level [21]. The MIDI instrument was developed to identify which determinants influence the actual use of an intervention [23]. Questions from the MIDI instrument were re-formulated to fit the aim of the semi-structured interviews in our study.

The topic list was used in a flexible way: within an iterative process of data collection and data analysis, if relevant, existing topics were adapted and new topics were added to the topic list based on the information obtained in previous interviews.

Semi-structured interviews were performed with 15 clients and 13 HCPs. Participants could choose where the interview would take place, at the mental health care setting or at the homes of the participants. All interviews took place near the end of the 12-months SMILE intervention period. Interviews with clients lasted 22 to 53 min with an average of 37 min, and interviews with HCPs 38 to 75 min with an average of 49 min. The interviews were digitally recorded and transcribed verbatim, anonymized and verified for accuracy. To check the credibility of the study, participants received a summary of their interview and were asked if they recognized the main themes described (member check) [24]. All but one of the HCP interviewees (92%) and 7 (47%) of the client interviewees responded for the member check, and agreed with the summary.

#### On-site observations of SMILE group sessions

We determined the fidelity to the structure of the SMILE intervention, the quality of the delivery of content and interaction within the group by using a standardised question form (Appendix III). This form was based on the form used during the STRIDE study [25]. During observations we did not register any specific data from clients.

### Data analysis

Quantitative descriptive data were analysed in STATA 16. The transcripts of the interviews were read and coded separately by two researchers (FW and JWdJ). MAXQDA 2018 software was used to facilitate qualitative data analysis [26]. Interviews were analysed using a thematic analysis as described by Braun and Clarke (2006) [20, 27]. This approach contains six phases: 1) familiarization with the data by reading and summarizing all transcripts; 2) generation of initial codes; 3) searching for themes; 4) reviewing if themes are valid; 5) defining and naming the themes, and 6) producing the report. Themes derived by both researchers were compared and discussed until consensus about the central themes was reached. Data collection stopped at the moment of data saturation. Agreement was reached on relevant themes within the research team and for each theme the most illustrating quotes were selected. Quotes were only adjusted if necessary for the readability of the final report, without affecting its content.

The content of the forms filled in during the observations was summarized. The most common observations were compiled into one document for subsequent analysis. We assessed fidelity to the intervention by rating (1) eleven components of the SMILE intervention, (2) the fidelity to the structure of the sessions (check-in, goal setting and physical activity part) and (3) the fidelity to the specific topic of the session, giving each of them scores of 0 (not implemented at all), 1 (partially implemented) or 2 (fully implemented). It should be noted that not all components were applicable to each session. The component was only scored if it was relevant for the specific session observed. Afterwards, all scores were summed up and divided by the total amount of scores, creating a mean score. In the end, results from the observations were integrated with the results from the interviews.

## Results

### Part 1: quantitative results

#### Execution of the intervention

The SMILE intervention consisted of 30 sessions per team over 1 year; a total of 330 sessions over the 11 intervention teams. At the end of the trial, 324 (98%) sessions were performed. Six sessions in one team were cancelled due to lack of personnel.

A total of 26 HCPs started performing the SMILE intervention. During the intervention, 10 of them (39%) dropped out, three HCPs were found as substitutes within the teams. The major reason for dropping out was changing jobs or discontinuation of work contract. Two teams had to substitute both trained HCPs.

#### Attendance during group sessions

In total 129 clients signed an informed consent to start with the SMILE intervention. However, eight clients never started with the intervention or did not have any baseline measurement for the trial, so ultimately 121 clients were included in the intervention. Group size varied from 7 to 16 clients. On average, clients were present at 15.8 of the 30 (53%) available sessions; on average 13.3 (55%) sessions out of 24 during the initial part of the intervention, and 2.6 (43%) sessions out of six during the maintenance sessions. After the start of the intervention, attendance at the sessions dropped to 71% after 1 month, 60% after 2 months and 50% after 3 months. After 15 sessions, 28% of the clients did not return to any of the sessions.

### Part 2: qualitative results

#### Characteristics of interviewees

Ten of the HCPs were female and three male. Their age ranged from 30 to 62 years. The majority of HCPs were registered nurses.

Six clients were male and nine were female. Age ranged from 30 to 61 years. Four clients gained weight (3.9 kg – 8.0 kg), one client experienced no change in weight, and ten participants lost weight (3.0 kg – 12.5 kg) after 6 months. Attendance to group sessions of interviewees varied from 3 to 30. Table 1 presents the characteristics of HCPs and clients.

**Table 1:** Participant characteristics

A. Characteristics of interviewed mental health care professionals					
**Nr**	**Gender**	**Age**	**Discipline**	**Total sessions given**	
HCP1	Male	60–69	Nurse	15	
HCP2	Female	40–49	Activity worker	20	
HCP3	Female	50–59	Nurse	23	
HCP4	Female	30–39	Nurse	24	
HCP5	Female	50–59	Nurse	30	
HCP6	Female	50–59	Nurse	28	
HCP7	Female	30–39	Social worker	27	
HCP8	Female	30–39	Psychologist	25	
HCP9	Female	30–39	Expert-by-experience	29	
HCP10	Female	30–39	Nurse	28	
HCP11	Male	50–59	Nurse	27	
HCP12	Female	50–59	Nurse	27	
HCP13	Male	40–49	Nurse	24	
B. Characteristics of interviewed clients					
**Nr**	**Gender**	**Age**	**Diagnosis**	**Weight change after 6 months**	**Total attendance**
C1	Male	50–59	Schizophrenia or other psychotic disorder	Gain	18
C2	Female	40–49	Borderline or other personality disorder	Loss	23
C3	Female	30–39	Borderline or other personality disorder	Loss	23
C4	Male	50–59	Schizophrenia or other psychotic disorder	Loss	14
C5	Male	40–49	Schizophrenia or other psychotic disorder	Gain	18
C6	Female	40–49	Depressive or bipolar disorder	Gain	3
C7	Female	50–59	Depressive or bipolar disorder	Loss	28
C8	Female	30–39	Schizophrenia or other psychotic disorder	Gain	11
C9	Male	40–49	Schizophrenia or other psychotic disorder	Loss	29
C10	Female	40–49	Depressive or bipolar disorder	Equal	23
C11	Female	50–59	Schizophrenia or other psychotic disorder	Loss	20
C12	Female	50–59	Post-traumatic stress disorder	Loss	21
C13	Female	30–39	Post-traumatic stress disorder	Loss	21
C14	Male	50–59	Schizophrenia or other psychotic disorder	Loss	30
C15	Male	60–69	Obsessive compulsive disorder	Loss	29

#### Interviews

All interviews were performed by FW and JWdJ between February 2019 and October 2019. Four main themes emerged from the interviews: 1) Positive appraisal of the SMILE intervention, 2) Suggestions for improvement of SMILE intervention 3) Facilitators of implementation and 4) Barriers of implementation. For each theme, different subthemes were identified for both clients and HCPs (Table 2). The most illustrative quotes linked to the (sub) themes are presented.

**Table 2:** Overview of themes and main results by clients and health care professionals

Theme	Subtheme	Clients	Healthcare professionals
**1)** Positive appraisal of the SMILE intervention	Clients enjoyed participating in the SMILE intervention	Clients perceived the intervention as useful, motivating and enjoyable. Clients had positive experiences with HCPs involved with SMILE.	Not applicable
	HCPs enjoyed conducting the SMILE intervention	Not applicable	HCPs enjoyed conducting the intervention and seeing positive results in clients.
**2)** Suggestions for improvement of the SMILE intervention	Tailoring the SMILE intervention to people with SMI	Not applicable	HCPs find the intervention suitable and interesting for all people with SMI. However, tailoring for the individual characteristics of patients is needed.
	No consensus on frequency of sessions of the SMILE intervention	Transition from weekly to monthly sessions is too big, however monthly sessions can have some benefits.	Believe transition from weekly to monthly sessions is too big, however workload was better during monthly sessions.
**3)** Facilitators of implementation	User-friendly handbook	Not applicable	Handbook was user friendly and provided detailed information which supported HCPs in conducting the intervention.
	Training of HCPs	Not applicable	Information regarding nutrition and other lifestyle related subjects were found important to learn during training.
**4)** Barriers of implementation	SMILE in combination with usual work	Not applicable	It was difficult to combine the SMILE activities with daily tasks. In order to conduct the intervention it is needed for (at least) two HCPs to be involved with SMILE in order to align work activities between HCPs. More time is needed to conduct the intervention.
	Lack of team and management support during implementation	A change in HCPs had a negative influence on the cohesion within the group.	In most teams HCPs felt no support from their other team members. They feel SMILE should be more of a priority within teams or management. A shortage of staff in general had a negative influence on the workload of HCPs during the delivery of SMILE.

#### Theme 1: positive appraisal of the SMILE intervention

In general, the SMILE intervention was positively evaluated. Clients and HCPs found being involved in the intervention enjoyable. Clients experienced the role of HCPs as group leaders during the intervention as positive.

##### Clients enjoyed participating in the SMILE intervention

Clients had positive experiences when participating in the SMILE intervention. They enjoyed attending the group sessions with other clients who shared the same goals, found the intervention program useful and informative, and had adequate learning experiences from participating in the group sessions. The SMILE intervention was perceived as complete and covering a substantial variety of relevant subjects. They found the design of the SMILE intervention appealing for receiving support for weight loss. Clients stated they were continuously motivated to set personal goals for lifestyle changes and it felt good for them to actively start working on their goals.

*“How the intervention was put together and what it was all about. And that it is important to set goals for yourself every time and that you try to achieve those goals.” (C7)*

Clients expressed positive experiences with regard to the HCPs role as group leaders during the execution of the intervention program. Clients valued the way HCPs were able to create a positive, safe and stimulating climate for realizing lifestyle changes. They also positively evaluated the knowledge and competences of the HCPs regarding lifestyle issues and lifestyle promotion.

*“They did very well. They had prepared it well every time and everything. And they also knew a lot of things and they had answers to many issues.” (C7)*

##### HCPs enjoyed conducting the SMILE intervention

The HCPs expressed their satisfaction with the SMILE intervention program. Their motivation to perform the intervention was strengthened due to the positive results they observed in clients, even when they did not lose weight. Because of the SMILE intervention, they were able to give structured attention to lifestyle promotion in clients with SMI, which they considered an important element in the overall treatment program for this client group. They expressed the opinion that this received too little attention in daily practice. They enjoyed being active with concrete lifestyle activities together with clients in a group setting and to guide people towards better self-care and self-management.

*“I really liked it, first of all. It is great to get people together who have the same goal. And it's also something that, generally, doesn’t get a lot of attention. And I notice the clients appreciate that we focus on this now.” (HCP8)*

#### Theme 2: suggestions for improvement of the SMILE intervention

##### Tailoring the SMILE intervention to people with SMI

HCPs mentioned that in principle the intervention was suitable and interesting for the participating clients, who all had overweight. In addition, it was mentioned that the intervention could also be of interest for clients who are not overweight. They stated that the SMILE intervention addressed a variety of subjects that were applicable for all clients who are interested in lifestyle changes and not only for those who are overweight or interested in weight loss. They valued the intervention because of its encompassing nature focused on improving lifestyle behavior and increasing quality of life, with elements such as the effect of healthy eating and exercising on body and mind, planning meals, mindful eating, stress and sleep quality. HCPs emphasized that sometimes it was needed to tailor the intervention to individual characteristics of patients, e.g., with respect to cognitive functioning.

*“We did not have to adjust the program itself, but we had to adapt the approach or repeat it more often. Even though it is not part of the program to look back, we did it anyway because there were certain people with mild intellectual disabilities. But, at the end, it was applicable for everyone who participated.” (HCP4)*

HCPs reported that it was sometimes difficult to present information and teach skills in a sufficiently comprehensible way for all participating clients. Some HCPs mentioned that the group diversity could also be beneficial, in particular for clients with lower learning capabilities, because they could learn from ideas and experiences of clients with higher cognitive functioning in the same group.

##### No consensus on changing frequency of sessions of the SMILE intervention

Views on the quantity of groups sessions of the SMILE intervention differed among clients and HCPs. One view was that the transition from weekly to monthly group sessions (after 6 months) was experienced as too abrupt for some clients. Both clients and HCPs mentioned that some of the clients experienced a lack of support and external motivation when changing from weekly to monthly sessions. Adding bi-weekly meetings as a transition or prolonging the weekly sessions was recommended. This could potentially help clients to better prepare for the maintenance phase.

*“Maybe I would do it once every two weeks for another few months and then reduce it to once a month.” (HCP11)*

The contrasting view from other clients and HCPs was that the transition to monthly sessions was experienced as positive. For some clients, the lower intensity of the SMILE intervention offered opportunities to focus on alternative treatment goals, such as starting a new job. For HCPs, the workload lowered substantially in the second phase of the SMILE intervention with monthly sessions, which was considered positive.

#### Theme 3: facilitators of implementation

HCPs particularly mentioned two facilitators that contributed to effective implementation of the SMILE intervention: the availability of a user-friendly handbook and the quality of the training.

##### User-friendly handbook

HCPs used the SMILE handbook and perceived it as user-friendly. The handbook provided detailed information on how to deliver the content of each session, with useful suggestions and background knowledge. For HCPs this meant they were not responsible for building the content and activities for the intervention.

*“Well, the handbook was nice to work with. So, I definitely needed the handbook, I couldn't have come up with it myself.” (HCP6)*

##### Training of HCPs

The SMILE training program was considered useful and informative by HCPs. The lifestyle related subjects, such as learning more about nutrition, cardiovascular risks of this client group, and the importance of physical activity to lower these risks, were considered important.

#### Theme 4: barriers of implementation

HCPs experienced a number of barriers when executing the SMILE intervention. They found it difficult to combine the intervention with usual tasks and sometimes experienced a lack of support from colleagues.

##### SMILE in combination with usual tasks

As the SMILE intervention took at least 2 h weekly during the first 6 months, HCPs found it difficult to combine the intervention with their other work responsibilities. They mentioned that their usual workload decreased very little while conducting the SMILE intervention. It was particularly difficult to combine the intervention with usual tasks when acute crisis situations occurred on the day the SMILE meetings were scheduled.

*“Well, if there was a crisis or something, I was fed up. Then I was like: gosh, I want to deal with that crisis, but I have to lead a SMILE session now. So, that was difficult for me. We held the program from 1 to 3 p.m. and I had a lot of phone calls after that. So, I think the setting could have been a bit less hectic.” (HCP3)*

We had anticipated on this barrier by involving at least two team members in conducting the intervention. However, some HCPs mentioned that even with two team members the intervention was difficult to accomplish without additional manpower.

*“I also thought it was quite difficult with the two of us. Especially during the weekly sessions. Then it is quite a lot.” (HCP10)*

Because of the intensive workload, HCPs often mentioned they lacked time for implementing the SMILE intervention. To be able to promote implementation of the SMILE intervention on a broad scale, more time would be needed.

*“More time. You really need time for this to unfold properly.” (HCP9)*

##### Lack of team and management support during implementation

There were differences between teams with respect to the intensity other members of the FACT-team were involved in the delivery of SMILE, aside from the trained HCPs. In some settings, SMILE was in the center of attention of the whole team. Here, HCPs felt sufficient support from their colleagues. However, in other settings, a lack of team involvement and support was experienced. HCPs suggested that a possible reason for this was the intensive workload experienced by other team members.

*“Several team members have other group sessions, and besides, everyone is just busy with their own individual appointments, of course, so everyone is busy.” (HCP7)*

The HCPs experienced that the SMILE intervention was not prioritized by other team members or by the management. Another reason mentioned for this lack of support was the overall staff shortage. This significantly hindered the implementation of new interventions, including SMILE.

*“And when you want to prepare it well … We have had staff shortages during the weekly sessions and then it is really difficult.” (HCP10)*

Staff shortage also influenced the clients participating in the intervention. In two teams both HCPs discontinued their job, hence their involvement in the SMILE intervention, and substitute HCPs were appointed to continue the intervention. Even though clients felt safe and at ease with the substitute HCP, it may have negatively influenced the cohesion within the group.

*“And the new group leader has taken over out of the blue as well as possible, but it is disastrous for a group, but also for the healthcare professionals...” (C2)*

HCPs emphasized that the whole team should be more involved in the intervention. Involving more team members, aside from the trained SMILE HCPs, would make the intervention an active topic of interest during meetings and daily care. In FACT-teams, all clients have a personal case manager who could play a more explicit role in integrating the content of the SMILE intervention in daily care.

*"So I think it also falls and stands with the case manager asking: “How are things going with SMILE?”. To mention and discuss that, when she sees them separately... Again, then it is more alive.” (HCP4)*

### Onsite observations of SMILE group sessions

In all 11 teams, at least one observation of a group session took place, and in five teams two sessions were observed, with 16 observations performed in total. To gain best insight into how the sessions were delivered in practice, fourteen observations took place after 2 months implementation during the weekly sessions, and two more during the monthly sessions. The data derived from the semi-structured interviews and onsite observations using the observation form (Appendix III) were highly consistent. Intervention component scores ranged from 1.3 to 2.0 with a mean of 1.7, which indicates an overall good fidelity to the components. The component ‘Aligning goals and action plans to stage of change’ scored lowest (1.3). This is because we performed only three observations where activities related to this component were specifically part of the session (as described in the handbook), of these three teams one team scored a 0. The components ‘Reducing portions and choosing alternatives’, ‘Developing core competencies of food choices’ and ‘Addressing mental health issues’ scored highest. Scores for individual components can be found in Appendix IV.

Throughout the observations, HCPs used the handbook during all on-site observations accurately. Regarding the structure of the sessions, the average scores for delivery of the ‘check-in’, ‘goal setting’ and ‘physical activity’ was 1.7, and the average score for ‘theme/content’ was 1.9. This indicates an overall high fidelity to the structure of the intervention. Only one team did not apply the structure of the sessions as prescribed in the handbook, as they wanted more freedom in tailoring the implementation to their clients’ needs. In terms of time-management, HCPs followed the structure of the handbook as closely as they could, but sessions occasionally took longer than the 2 h intended. In most teams, the ‘check-in’ took more time than the prescribed 10 to 20 min. As the theme/content of the session occasionally took longer, there was sometimes less time available for the physical activity component than the prescribed 20 to 30 min). In addition, weigh-ins were not always structurally performed in some teams. In all teams we observed a lively interaction between clients, and between clients and HCPs. The finding from the interviews that both HCPs and clients enjoyed coming to the sessions and experienced it as a fun and useful activity, including the social aspect of it, was strongly confirmed in the observations.

In the interviews, HCPs mentioned the need to adapt the delivery of the SMILE content for some group members with (mild) intellectual disabilities. This was confirmed by the observations. We identified a wide disparity of intellectual abilities between clients within the groups. During all observations, HCPs actively included all clients in the interaction within the group by adapting their manner of explaining content to each specific situation, to make the content easier to understand. To complement this, some HCPs gave extra personal attention and time to clients with lower cognitive abilities after the session ended.

## Discussion

To the best of our knowledge, this is the first study analyzing implementation feasibility of a one-year group-based lifestyle intervention in Dutch FACT-care in a real-life setting. This study reports the results of the process evaluation exploring the experiences and perceptions of clients with SMI and HCPs regarding the implementation of the SMILE intervention in a real-life setting. The study helps to understand the factors that affect implementation of such lifestyle interventions in outpatient mental health care.

In our discussion we will make use of the different components of the RE-AIM model: Reach, Effectiveness, Adoption, Implementation and Maintenance [21].

### Reach

The component Reach of the RE-AIM model refers to the absolute number, proportion, and representativeness of individuals who are willing to participate in the SMILE intervention. In our study, the Reach component was achieved to a relatively high degree. Most mental healthcare institutes that we invited to participate in the study were interested. Four mental healthcare organization approached were not interested, because they had other priorities within their organization and expected insufficient available time for FACT-teams to participate. We managed to include 121 clients in the intervention. We also managed to deliver the intervention in multiple FACT-teams from different mental healthcare organizations and regions throughout the Netherlands. Given the interest in the intervention and the study, we were able to include more teams than we expected. Several factors may have influenced the recruitment process. We noticed big differences between teams regarding how many potential clients they recruited for the intervention. Teams that mentioned it would be difficult to fill a group due to lack of motivated clients often had smaller groups. While the specific reason for this is unknown, the phenomenon of ‘self-fulfilling prophecy’ may have occurred. In addition, we noticed that teams in which other team members did not cooperate sufficiently during recruitment ended up with smaller groups as well. In clinical practice, HCPs may be pessimistic about the ability of people with SMI to embrace lifestyle changes and to achieve significant health changes [28]. For future research and practice, we recommend being aware of possible treatment pessimism in HCPs that influences the motivation of both HCPs and clients to participate in a lifestyle intervention.

### Effectiveness

Effectiveness refers to the impact of the SMILE intervention on the outcomes. The main outcomes of the RCT regarding the effectiveness of the intervention will be published elsewhere. We found important information in this process evaluation that could improve implementation of the SMILE intervention. The SMILE intervention was overall evaluated as enjoyable for clients and HCPs. This can be an important factor that influences the motivation to continue with the intervention for 1 year. Moreover, facilitating factors such as the use of a pragmatic handbook for HCPs will facilitate implementation of the SMILE intervention.

### Adoption

Adoption refers to the proportion and representativeness of people who deliver the intervention and who are willing to initiate the SMILE intervention, and why. The setting in which the SMILE intervention was delivered was not always optimal for successful delivery of the intervention. HCPs experienced difficulties in being able to integrate delivery of the intervention in usual care and a lack of support from team members. Relatively many HCPs dropped out during the phase of the study in which the intervention was delivered (first 6 months). This is in line with the STRIDE study that also encountered a high rate of staff turnover. In the SMILE study, as in the STRIDE study, a co-leader model was used to diminish the risk of lowering the dose of delivery. Discontinuity of care in vulnerable populations such as this could be problematic [29]. This population may have a greater need for good continuity of care due to illness symptoms (e.g., negative symptoms), memory problems and cognitive deficits [29]. Therefore, it is important for participants to be comfortable with their HCPs without discontinuity in the relationship, and with sufficient time for establishing helpful HCP-client relationships [29].

### Implementation

Implementation entails the fidelity to the SMILE intervention and used resources (e.g., time). Results showed that implementing an extensive one-year lifestyle intervention is feasible in this ambulatory setting. Attendance to group sessions in the SMILE study (55% during the weekly sessions and 43% during monthly sessions) would ideally be higher, but was similar to the STRIDE study (60 and 45% respectively) [18]. The SMILE handbook was adequately used during sessions. The handbook appeared to be an extensive and useable tool that facilitated the delivery of the intervention as intended. The average scores (1.7 out of 2.0 maximum score) on implementation of the components of the intervention program are in line with the STRIDE study [25]. HCPs mentioned the need to adapt the manner of delivery to some clients. This is also in line with the publications of the STRIDE study [25]. Moreover, a Dutch lifestyle study performed in a similar setting as the SMILE study (the LION study), also reported that some clients may need more support than others [16]. This emphasizes and confirms the importance of tailoring a lifestyle intervention in this diverse population [25, 28]. However, it should be noted that implementing lifestyle interventions in people with SMI remains challenging [14, 28, 30]. In our study, HCPs experienced the need to tailor the intervention to the individual clients within the same group, given the varying levels of - for example - learning capabilities. This is particularly challenging for the HCPs to achieve, but a significant factor contributing to the effectiveness of the intervention. This is in line with literature, where cognitive deficits, negative symptoms and a lower health literacy are recognized as challenges for the implementation of lifestyle interventions in this population [28, 31–35]. In the end, participating in a lifestyle intervention can be experienced as complex or difficult by a part of the clients [28, 36]. To overcome these barriers, studies suggest that more time and efforts are needed to tailor lifestyle interventions to the patients’ needs and competencies [28, 31, 37–40].

We highly recommend future research to include a structured process evaluation to gain insight into the actual delivery in practice and the experienced barriers and facilitating factors, preferably by using different data sources such as quantitative and qualitative data.

### Maintenance

Maintenance evaluates the sustainability of the implementation of the intervention on both the individual and organizational level. To be able to institutionalize and secure the implementation of the SMILE intervention on the long term, our process evaluation has shown the importance of having multiple members in a team who are dedicated to investing time in a lifestyle intervention, and the commitment of the (management of the) mental healthcare organizations involved in terms of support with (financial) resources. Training multiple staff members on lifestyle related subjects, and giving them specific responsibilities within the team in this area, is therefore highly recommended for future practice and research [25]. We found that management did not always prioritize or stimulate implementation of the intervention (other than giving HCPs permission to deliver the intervention), with the consequence that the whole delivery was up to the two HCPs involved with the intervention. This limits the possibilities for sustainable integration of the intervention in practice. Organizational determinants related to the implementation and maintenance of a lifestyle intervention have previously been described as being predictive for its success [28, 36, 41]. As previous studies have already suggested, there is a need for mental healthcare organizations to actively put the subject of lifestyle on the agenda and facilitate time and resources to enable sustainable implementation [16].

## Strengths and limitations

### Strengths

In the Netherlands and many other countries, there is an increase of ambulatory treatment and the concomitant decrease of inpatient treatment for people with SMI. The Dutch FACT setting, providing such ambulatory treatment, is a relevant setting to learn more about the implementation of an extensive lifestyle intervention for people with SMI. To date, few process evaluations have been published about the implementation of lifestyle interventions within outpatient settings. In this regard, the present study is valuable for both practice and research. We were able to gain insight into experiences and perceptions of clients and HCPs in a real-life setting which can help us understand how a lifestyle intervention works in daily practice. This is highly relevant, as typically trials are often performed in ideal conditions that do not always reflect typical practices related to the implementation of the intervention in real-life conditions.

Furthermore, a strength is that we applied data triangulation using several sources of data in order to increase the credibility of this study: quantitative descriptive data about the implementation, interviews focusing on perceptions from two different perspectives (HCPs and clients), and observational data. This enabled us to confirm the results from the interviews with objective data.

Quality was also ensured by the use of two independent analysts, the systematic development of codes, and achieving a high degree of data saturation of information after coding the interviews. By including participants from different settings spread across the country the transferability of our data is also high, though limited to ambulatory mental health settings.

Finally, we made use of the theoretical frameworks RE-AIM and MIDI in this process evaluation. By using these validated and internationally well-known frameworks, we were able to conduct a thorough analysis of the implementation process, including experienced barriers and facilitators.

### Limitations

A limitation of the process evaluation is that all interviews and observations had to be conducted by researchers who were also involved in the RCT on the effectiveness of the SMILE intervention, which might have influenced the objectivity of the analysis. To regulate this potential source of bias, the researchers discussed the outcomes of both observations and the interviews repeatedly during research meetings with collaborators who were not directly involved in the data collection of the SMILE study.

A second limitation is that within this study only FACT-teams joined the study that were motivated to participate, which may affect transferability of the study results.

## Conclusions

This study finds that the SMILE intervention was feasible and well experienced by clients and HCPs. There was an overall good fidelity to the intervention components. We identified essential aspects that can both hinder or stimulate effective implementation and maintenance of the lifestyle intervention. The positive attitude of both HCPs and clients towards the SMILE intervention was a central facilitator for implementation. Main barriers were time management and a lack of team and management support. Researchers, HCPs and mental healthcare organisations need to be aware of the importance of tailoring the lifestyle intervention to the individual characteristics and motivations of individuals with SMI. The results can be used to further develop dissemination and implementation of the SMILE intervention in mental healthcare.

## Supplementary Information

**Additional file 1.**

## Data Availability

The data that support the findings of this study are not publicly available, because that is inconsistent with the informed consent. However, data are available from the corresponding author on reasonable request and with permission of the VU Amsterdam.

## Abbreviations

HCPs: Healthcare professionals
MIDI: Measurement instrument for determinants of innovations
SMILE: Severe mental illness lifestyle evaluation
SMI: Severe mental illness
RE-AIM: Reach Effectiveness - Adoption Implementation Maintenance
FACT: Flexible Assertive Community Treatment
