# Supplementary material for: Implementation of a lifestyle intervention for people with a severe mental illness (SMILE): a process evaluation
Source: BMC Health Serv Res. 2022 Jan 5;22:27. doi: 10.1186/s12913-021-07391-3 (PMC8729040; doi:10.1186/s12913-021-07391-3)
Supplement: Supplementary file 1 — Additional file 1. [file 12913_2021_7391_MOESM1_ESM.docx]

# Appendix I Overview of group sessions

| **Weekly sessions** | **Content of session** |
| --- | --- |
| Session 1 | Welcome and introduction to SMILE |
| Session 2 | Portion sizes |
| Session 3 | Energy balance and goal setting |
| Session 4 | Breakfast, physical activity and regular eating |
| Session 5 | Working on a healthy eating pattern |
| Session 6 | Eating healthy on a budget |
| Session 7 | Progress check |
| Session 8 | Meal planning |
| Session 9 | Your environment and social support |
| Session 10 | Adverse effects of medication and weight gain |
| Session 11 | Sleeping behaviour and coping with stress |
| Session 12 | Progress check and tackling negative thinking |
| Session 13 | Eating consciously |
| Session 14 | Over-eating and emotional eating |
| Session 15 | Eating out |
| Session 16 | Importance of physical activity |
| Session 17 | Meal planning and portion sizes |
| Session 18 | Progress check and problem solving |
| Session 19 | Social support |
| Session 20 | Pitfalls |
| Session 21 | Stagnation of progression |
| Session 22 | How to maintain weight loss |
| Session 23 | Coping with changes in mental health status |
| Session 24 | Celebrating successes |
| **Monthly sessions** |  |
| Session 25-30 | Recap of former topics and free input |
|  | |

# Appendix II Topic lists

*Topic list for interviews with clients with SMI*

| **General** |
| --- |
| What did you think of the SMILE intervention? |
| **Participation, suitability, what does the group know** |
| *Key questions*  Why have you decided to participate in this intervention?  What did you expect from the intervention?  What did you find interesting that made you return to the sessions?  You did not come anymore, how did that come about? |
| **Adoption of SMILE intervention** |
| *Key questions*  In the intervention, you have had guidance from [XX] and [XX], how did they do it? |
| **Group sessions** |
| *Key questions*  What did you like about the intervention?  First you got weekly sessions, what did you think of the weekly sessions?  And when the monthly sessions came?  In each session there was a reflection or evaluation, what did you think of that?  You have received handouts in the intervention. What did you think of these handouts?  How did you use it? |
| **The group** |
| *Key questions*  How did you feel in the group? How did that come about?  If it were up to you, what would the group look like to you? |
| **Maintenance** |
| *Key questions*  What could we do better? Did you miss any topics?  Would you recommend the intervention to others?  Are you going to miss it?  What would help you stay on the path where you are now? |
| **Final question**  Is there anything else you want to talk about? |

*Topic list for interviews with healthcare professionals*

| **General** |
| --- |
| How did you like to give SMILE? |
| **Participation, suitability, what does the group know** |
| *Key questions*  What are the main reasons for you to run the SMILE intervention?  Do you think the intervention is in line with the SMI target group?  Clients could only participate in the intervention if they were overweight. To what extent were the participants aware of their overweight? To what extent were participants aware of unhealthy habits? |
| **Effectiveness** |
| *Key questions*  What has changed in behavior during the SMILE intervention?  Has the SMILE intervention helped people change their behavior?  How is it that SMILE works for some people and not others?  What does it take to make SMILE successful with those it hasn't helped?  When is the intervention effective?  What are the main successful elements / components of SMILE?  What influence did the social environment / home situation have on the participants? |
| **Group sessions** |
| *Key questions*  Which group sessions have you remembered the most?  What was the best element and what was not? |
| **The group** |
| *Key questions*  How did the group interact with each other? What was the group dynamics like?  How did you deal with the group dynamics? |
| **Maintenance** |
| *Key questions*  Would you like to start a new SMILE group right now?  Why / why not?  Would it be possible to start a new group?  How can SMILE be improved?  What would be required to implement the intervention within FACT?  How would you ideally view lifestyle intervention in people with SMI?  What advantages and disadvantages did the intervention have? |
| **Final question**  Have we forgotten things you want to talk about? |

# Appendix III Observation form

**Observations of the group sessions**

Cohort #: ____ Session #: ____ Date: _____/_____/______ Observer:_________________

**Part 1: Strategies**

For each of the strategies below, assess whether they are relevant to this session. If so, please state the extent to which the strategy was implemented. Use the comments if necessary.

1. **Self-monitoring of nutrition and physical activity (tracking and awareness)
   Relevant?** Yes / No

**If so: to what extent implemented:** not at all partially completely

**Remarks:**

1. **Setting personal goals regarding eating and exercise patterns
   Relevant?** Yes / No

**If so: to what extent implemented:** not at all partially completely

**Remarks:**

1. **Moderately reducing calorie intake
   Relevant?** Yes / No

**If so: to what extent implemented:** not at all partially completely

**Remarks:**

1. **Reducing portion sizes and replacing high-calorie products with lower-calorie products.
   Relevant?** Yes / No

**If so: to what extent implemented:** not at all partially completely

**Remarks:**

1. **Increase the intake of vegetables, fruit and fiber.**

**Relevant?** Yes / No

**If so: to what extent implemented:** not at all partially completely

**Remarks:**

1. **Increasing physical activity**

**Relevant?** Yes / No

**If so: to what extent implemented:** not at all partially completely

**Remarks:**

1. **Identifying situations associated with undesirable lifestyle behaviors and formulating and practicing plans to deal with these situations.**

**Relevant?** Yes / No

**If so: to what extent implemented:** not at all partially completely

**Remarks:**

1. **Mapping progress of weight loss and behavioral change.
   Relevant?** Yes / No

**If so: to what extent implemented:** not at all partially completely

**Remarks:**

1. **Developing core competencies of food choices**

**Relevant?** Yes / No

**If so: to what extent implemented:** not at all partially completely

**Remarks:**

1. **Aligning personal goals and action plans to the needs and stage of change of the participant.
   Relevant?** Yes / No

**If so: to what extent implemented:** not at all partially completely

**Remarks:**

1. **To draw attention to the influence of mental health on lifestyle changes, including: medication, sleep coping with symptoms, etc.
   Relevant?** Yes / No

**If so: to what extent implemented:** not at all partially completely

**Remarks:**

**Part 2. Check-in, goal setting and physical activity (in all sessions)**

To what extent have the group leaders applied this?

**A. Check-in:**  Not at all Partly Thorough / Completely

**B. Setting goals:** Not at all Partly Thorough / Completely

**C. Physical Activity:**  Not at all Partly Thorough / Completely

**Remarks:**

**Part 3. Questions specific to the topic of the session**

Answer the following questions related to the topics listed in the Handbook for the specific session you are observing.

1. **To what extent have the group leaders discussed the topic of the session? (Has the planned content of the session been discussed?)**

Not at all Partly Thorough / Completely

**Remark:**

**If not, was the deviation from the planned content appropriate?**

Yes No

**Remark:**

1. **How did the participants react to the session content?**

Perceived attention:

Understanding:
Enthusiasm and involvement:
Observation about how useful the participants found the material:

**Remark:**

**Part 4. Quality.**

Describe the quality of the session in general:

# Appendix IV Scores of components in observations

| **Component** | **Score (range 0-2)** |
| --- | --- |
| Self-monitoring of nutrition and physical activity | (1.8) |
| Personalized diet and physical activity plans | (1.6) |
| Moderately reducing calorie intake | (1.7) |
| Reducing portions and choosing alternatives | (2.0) |
| Increase the intake of vegetables, fruit and fiber | (1.6) |
| Increasing physical activity | (1.6) |
| Planning for high-risk situations | (1.6) |
| Mapping progress of weight loss and behavioral change | (1.4) |
| Developing core competencies of food choices | (2.0) |
| Aligning goals and action plans to stage of change | (1.3) |
| Addressing mental health issues | (2.0) |
